# Safety of outpatient non-upper airway surgery for patients with obstructive sleep apnea in ambulatory surgical centers: A systematic review

**DOI:** 10.1371/journal.pone.0326704

**Published:** 2025-07-07

**Authors:** Muaaz Asghar, Kenny Pang, Mauz Asghar, Brian Rotenberg

**Affiliations:** 1 College of Medicine, University of Saskatchewan, Saskatoon, Saskatchewan, Canada; 2 Asia Sleep Centre, Singapore, Singapore; 3 Department of Otolaryngology, Head and Neck Surgery, Schulich School of Medicine and Dentistry, London, Ontario, Canada; Cleveland Clinic, UNITED STATES OF AMERICA

## Abstract

**Objective:**

The systematic review aims to determine the safety of conducting non-upper airway surgery in an ambulatory surgery center (ASC) for OSA patients.

**Data sources:**

A comprehensive search was conducted from MEDLINE, Embase, CENTRAL, and Scopus from inception through February 2023.

**Review methods:**

Studies including non-upper airway surgery done in ASC settings were identified. Risk of bias was assessed using the Murad Tool and Newcastle-Ottawa scale. Primary outcomes were 24 hour complications and unplanned admission rates.

**Results:**

From 9313 studies, 13 non-OSA studies with 31,200 OSA participants and 318,709 non-OSA participants were identified. Severe complications were rare and tended to occur within the first 4 hours of the postoperative period. While a majority of smaller scale studies found no significant difference in unplanned admissions, large scale studies with multivariate analysis find OSA to be an independent risk factor for unplanned admission and 30-day complications. However, large scale ASC studies have found that with proper selection and perioperative interventions, OSA patients can undergo outpatient surgery at ASCs safely.

**Conclusions:**

OSA patients with mild or controlled comorbidities can safely undergo ambulatory non-OSA surgery in ASCs.

**Other:**

The protocol for this review was registered with the PROSPERO database (Registration number: CRD42023415162).

## Introduction

Obstructive sleep apnea (OSA) is the one of the most common disorders, affecting 6–19% of the general population, and up to 49% of the elderly population. OSA has severe clinical consequences as it has been linked to heart disease, stroke, and diabetes [1–4]. The lack of bony support and external factors such as body mass index (BMI) and age can cause airway collapse along the soft palate to the larynx [5]. These collapses lead to apneas and awakenings during sleep, interrupting normal sleep structure, leading to the aforementioned diseases.

In the context of inpatient surgeries, OSA has been identified as a significant risk factor for major postoperative cardiopulmonary complications, with an associated increased risk ranging from 56% to 91% [6,7]. As such, patients with OSA have been seen as a significant contraindication to outpatient surgery. However, a consensus statement from the Society for Ambulatory Anesthesia has recommended that patients with well-controlled OSA and comorbidities can undergo outpatient hospital surgery [8]. As the need for outpatient surgery has grown over the last two decades [9], ambulatory surgery centers (ASCs), especially free-standing ASCs, were formed to facilitate this growth [10,11]. The use of ASCs are also a benefit to our burdened healthcare system as it has been shown to be cost-saving [12,13]. Despite the growing popularity of ASC, there has been no statement or review regarding the safety of patients with OSA undergoing surgery in these facilities.

There has been a recent meta-analysis that has found OSA is a significant risk factor for unplanned admissions and severe complications especially for orthopedic surgery [14]. However, most of the studies used in the analysis were in hospital outpatient departments. ASCs have different resources to that of a hospital outpatient setting. The lack of a closeby intensive care unit and limited resources means that severe complications should be limited. Reducing the risk of unplanned admission is imperative as hospital transfers and admissions would be more costly to patients. As such, the selection of patients for ASCs differs from that of a hospital setting and requires its own review.

There are no systematic reviews analyzing the effect of OSA on the safety in an ASC environment. Therefore, this review aims to answer the question: Can patients with OSA safely undergo non-upper airway surgeries in an ASC?

## Methods

A systematic review was conducted in accordance with the Preferred Reporting Items for Systematic Reviews and Meta-Analyses statement (PRISMA) [15]. A comprehensive search of Ovid-MEDLINE, Ovid-Embase, Cochrane, and Scopus was conducted using keywords including database-specific keywords from inception to February 2024 with the assistance of a librarian (S1–S4 Tables).

Title and abstracts were deduplicated by Rayyan (https://www.rayyan.ai/) and were then independently examined by two authors (MA, MUA). Non-upper airway surgeries were analyzed whether they were performed at an ASC or the hospital setting.

Once relevant abstracts were identified, full text-articles were assessed. They were compared to a specific checklist of inclusion and exclusion criteria. (S1 File and Table 1) Any discrepancies were discussed and resolved via consensus. Initial data collection was conducted by one author (MUA) and was independently confirmed by another (MA). The primary outcomes are 24-hour complications and unplanned admissions. The following characteristics were also collected for each study: first author, year, study design, number of outpatients/inpatients, number of patients with/without OSA, participant characteristics, selection and admission protocol, type of surgery performed, 30-day postoperative complications, unplanned admission causes, follow-up length, factors for unplanned admissions or postoperative complications, and surgical setting.

**Table 1:** Inclusion and exclusion criteria of OSA and non-OSA surgery groups.

Population	≥10 OSA patients identified with a questionnaire, PSG, self report, or preoperative records who are 18 years or older that are intended to have outpatient same-day discharge surgery.
Intervention	Any non-upper airway surgery not used to treat OSA as its primary purpose excluding cardiac or vascular surgery.
Comparison	Not necessary but patients without OSA identified with a questionnaire, PSG, self report, or preoperative records who are 18 years or older that are intended to have outpatient surgery.
Outcome	Primary outcome is unplanned admissions 24-hour complications. Secondary outcomes are 24-hour complications, 30 day complications/readmission/ER visits and mortality rate.
Study Design	Retrospective or prospective studies case series, cohort, case-control, and RCTs
Exclusion Criteria	Conference abstracts, pediatric population, reviews, questionnaires, Local anesthetic with sedation, expert opinions, recommendations, animal subjects, non-English

The risk of bias assessment was performed by a single author (MA). Newcastle Ottawa Scale (NOS) and Murad et al. was used to look at cohort/case-control studies and case series studies, respectively [16–18] (S2–S4 Files). For NOS, 8–9 score was considered high quality, 6–7 was considered moderate quality, and 5 or less was considered low quality. The protocol for this review was registered with the PROSPERO database (Registration number: CRD42023415162).

Weighted averages were calculated for unplanned hospital admission rates, 24 hour and 30-day postoperative complication rates. Median was used for the calculation of the weighted average when a mean was not present.

## Results

**Fig 1:**
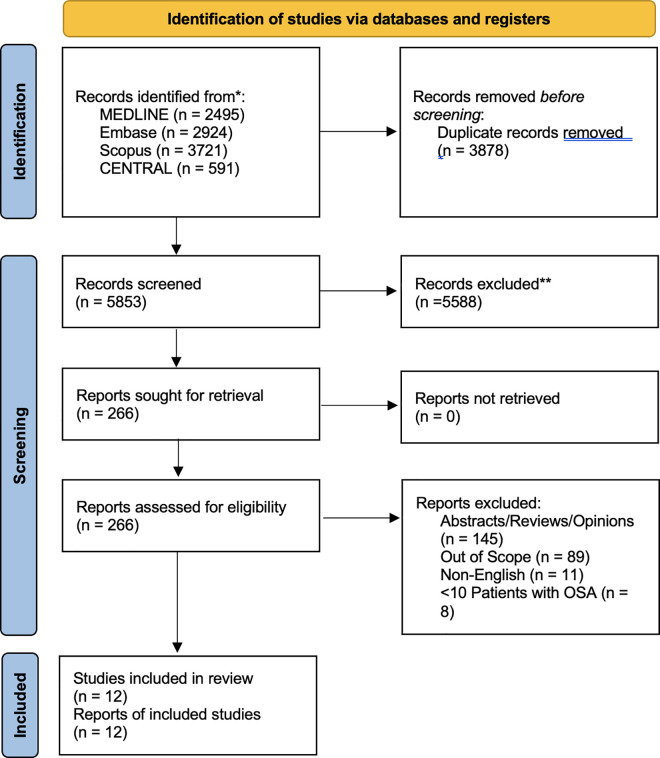
PRISMA flow diagram.

Our searches yielded 9731 articles, from which 3798 were found to be duplicates and removed. Following a thorough review of titles and abstracts, 266 full-text articles were examined. Ultimately, 12 studies met the inclusion criteria [19–30]. Fig 1 uses the PRISMA flow diagram to illustrate the search and selection process (S1 Data). Tables 2–8 presents characteristics, outcomes, and findings of the included studies (S2 Data).

**Table 2:** Study characteristics for hospital settings.

Study	Patients WITH OSA						Patients WITHOUT OSA					
	# of Patient	Age	BMI	Male (%)	Severity of OSA	Comorbidities	# of Patients	Age (y)	BMI	Male (%)	Severity of OSA	Comorbidities
Bryson, 2012 [20]	674	N/A	N/A	63.5	Mild AHI 5–15 = 23% Moderate AHI 16–30 = 10% Severe AHI > 30 = 11%	N/A	873	N/A	N/A	35.2	N/A	N/A
Dupanovic, 2017 [22]	76	N/A	N/A	N/A	N/A	N/A	125	N/A	N/A	N/A	N/A	N/A
Hudson, 2018 [23]	66	46.1 ± 11.1	29.3 ± 3.6	90.9	Mean REI = 9.1 ± 11.7	Hypertension: 31.8% Hyperlipidemia: 27.3% GERD: 18.2% Depression: 4.5% Hypothyroid: 4.5% Migraine: 6.1% Asthma: 6.1%	100	35.4 ± 9.6	27.2 ± 4.0	55.0	N/A	Hypertension: 8% Hyperlipidemia: 7% GERD: 10% Depression: 5% Hypothyroid: 6% Migraine: 2% Asthma: 2%
Jackson, 2023 [24]	205	N/A	N/A	N/A	N/A	N/A	433	N/A	N/A	N/A	N/A	N/A
Kleipool, 2023 [25]	49	43 ± 12	42 ± 3	26.5	N/A	Hypertension: 12.2% Diabetes: 10.2% Dyslipidemia: 8.2%	N/A	N/A	N/A	N/A	N/A	N/A
Masaracchia, 2018 [26]	7761	55.8 ± 11.1	N/A	67.7	N/A	Heart Disease: 4.4% Cerebrovascular disease: 0.86% COPD: 22.2% Liver Disease: 0.08% Diabetes: 32.1% Renal Disease: 2.5% AIDS: 0.03% Cancer: 0.45%	121171	48.2 ± 16.1	N/A	60.3	N/A	Heart Disease: 1.5% Cerebrovascular disease: 0.29% COPD: 9.5% Liver Disease: 0.10% Diabetes: 11.3% Renal Disease: 0.62% AIDS: 0.03% Cancer: 0.24%
Sabers,2003 [28]	234	57.0 ± 12.8	35.5 ± 7.2	73.1	RDI: 40.2 ± 30.2	Hypertension:43.6% Coronary artery disease: 11.1% Heart Failure: 3.0% Dysrhythmias: 5.6% Other cardiovascular disease: 1.7% Diabetes: 11.0% COPD: 3.9% Asthma: 6.8% Cerebrovascular disease: 1.3% Renal Disease: 1.3% Liver Disease: 0.4%	234	56.9 ± 13.1	33.7 ± 7.2	73.1	N/A	Hypertension:26.1% Coronary artery disease: 9.8% Heart Failure: 1.7% Dysrhythmias: 3.0% Other cardiovascular disease: 0.4% Diabetes: 5.2% COPD: 3.9% Asthma: 6.4% Cerebrovascular disease: 1.3% Renal Disease: 0.9% Liver Disease: 1.3%
Scotcher, 2023 [30]	23	N/A	N/A	N/A	N/A	N/A	N/A	N/A	N/A	N/A	N/A	N/A

**Table 3:** Study characteristics for ASC settings.

Study	Patients WITH OSA						Patients WITHOUT OSA					
	# of Patient	Age	BMI	Male (%)	Severity of OSA	Comorbidities	# of Patients	Age	BMI	Male (%)	Severity of OSA	Comorbidities
Berend, 2018 [19]	171	N/A	N/A	N/A	N/A	N/A	1301	N/A	N/A	N/A	N/A	N/A
Melton, 2020 [27]	21190	N/A	N/A	N/A	N/A	N/A	190199	N/A	N/A	N/A	N/A	N/A
Stierer 2010 [29]	94	N/A	N/A	N/A	N/A	N/A	2045	N/A	N/A	N/A	N/A	N/A

**Table 4:** Study characteristics for studies in both hospitals and ASC setting.

Study	Patients with OSA						Patients WITHOUT OSA					
	# of Patient	Age	BMI	Male (%)	Severity of OSA	Comorbidities	# of Patients	Age	BMI	Male (%)	Severity of OSA	Comorbidities
Christian, 2019 [21]	97	N/A	N/A	N/A	N/A	N/A	604	N/A	N/A	N/A	N/A	N/A

**Table 5:** Study outcomes for studies in hospital settings.

Study	Surgery Performed	Patients WITH OSA				Patients WITHOUT OSA			
		Admission Rate (%)	24 hour Postoperative complication rate (%)	30 day complication rate (%)	Mortality Rate (%)	Admission Rate for patients (%)	24 hour postoperative complication rate (%)	30 day complication rate (%)	Mortality Rate (%)
Bryson, 2012 [20]	All excluding Upper Airway surgery	6.7	16.0	0.3% readmissions	0.0	5.6	13.1	N/A	N/A
Dupanovic, 2017 [22]	Gastric banding	23.7	N/A	0% readmissions	0	22.4	N/A	0% readmissions	0
Hudson, 2018 [23]	Orthopedic extremity surgery	18.2	34.8	0.0% readmissions	0.0	12.0	38.0	0.0% readmissions	0.0%
Jackson 2023 [24]	Sleeve gastrectomy	47.3	N/A	N/A	N/A	26.3	N/A	N/A	N/A
Kleipool, 2023 [25]	Laparoscopic Roux-en-Y Gastric Bypass	8.2	6.1	8.2 complications, 14.3 revisits, 8.2 readmissions	0.0	N/A	N/A	N/A	N/A
Masaracchia, 2018 [26]	Shoulder arthroscopy	<0.1	N/A	4.10 complications, 0.21 readmissions	<0.1	<0.1	N/A	1.22 complications, 0.20 readmissions	<0.1
Sabers, 2003 [28]	All excluding ENT surgery	23.9	2.1	N/A	N/A	18.8	1.3	N/A	N/A
Scotcher, 2023 [30]	Shoulder surgery	0.0	0.0	0.0	0.0	N/A	N/A	N/A	N/A

**Table 6:** Study outcomes for studies in ASC settings.

Study	Surgery Performed	Patients WITH OSA				Patients WITHOUT OSA			
		Admission Rate (%)	24 hour Postoperative complication rate (%)	30 day complication rate (%)	Mortality Rate (%)	Admission Rate for patients (%)	24 hour postoperative complication rate (%)	30 day complication rate (%)	Mortality Rate (%)
Berend, 2018 [19]	Hip Arthroplasty	4.1	N/A	N/A	N/A	3.4	N/A	N/A	N/A
Melton, 2020 [27]	All surgeries	0.14	N/A	N/A	N/A	0.11	N/A	N/A	N/A
Stierer 2010 [29]	All surgeries	0	N/A	N/A	N/A	0.5	N/A	N/A	N/A

**Table 7:** Study outcomes for studies in hospital and ASC settings.

Study	Surgery Performed	Patients WITH OSA				Patients WITHOUT OSA			
		Admission Rate (%)	24 hour Postoperative complication rate (%)	30 day complication rate (%)	Mortality Rate (%)	Admission Rate for patients (%)	24 hour postoperative complication rate (%)	30 day complication rate (%)	Mortality Rate (%)
Christian, 2019 [21]	Shoulder arthroscopy	59.8	N/A	N/A	N/A	29.0	N/A	N/A	N/A

**Table 8:** Relevant findings of included studies.

Study	Selection criteria; Discharge criteria	Cause for admission for OSA population and; non-OSA population	24-hour Specific Complications for OSA population and; non-OSA population	Relevant findings of study
Berend	Functionally independent, excluded if they have non-optimizable comorbidities like: congestive heart failure, valvular disease, severe chronic obstructive pulmonary disease (COPD), home oxygen use, untreated sleep apnea with BMI > 40, severe renal disease, cerebrovascular incident, organ transplant; surgeon/anaesthesiologist	N/A	N/A	ASC study that found no statistically significant difference between OSA and non-OSA, uses a defined criteria, admissions extracted are only for medical causes as it was the only extractable data within the study.
Bryson	Need a PSG on file, need to be treated with PAP for sleep apnea, surgeon/anaesthesiologist judgment; surgeon/anaesthesiologist judgment	N/A	N/A	Controlled for undiagnosed sleep apnea, no statistically significant difference found between OSA and non-OSA population for admissions or complications, both populations had higher admission rate than general population. Age, BMI, severity of OSA and male gender were not correlated with admission. Only study with all complications and uses estimated complication rate
Christian	surgeon/anaesthesiologist; surgeon/anaesthesiologist	N/A	N/A	High admission rate for patients with OSA
Dupanovic	surgeon/anaesthesiologist; surgeon/anaesthesiologist	N/A	N/A	Found no significant difference between OSA and non-OSA populations
Hudson	surgeon/anaesthesiology, ASA physical status I-II with no pregnancy or drug/alcohol chronic use, Age < 65; hospital discharge criteria, surgeon/anaesthesiologist	41.7% Respiratory monitoring, 25% Pain, 8.3% Antibiotics; 41.7% Pain, 16.7% Respiratory monitoring, 16.7% Pain	28.9% Supplemental O2 10.6% Tachycardia, 3.0% Bradycardia; 22% Supplemental O2, 17% Tachycardia 6% Bradycardia 4% Other arrhythmias	Gave a respiratory polygraphy to each patient beforehand, Anaesthesia team only learned about OSA diagnosis before the study, no significant difference in complications or admissions, small sample size; respiratory complications defined as supplemental oxygen use, reintubation, or airway obstruction requiring ventilation
Jackson	BMI < 60, Age < 65; <100 bpm, systolic BP> 90, oral tolerance, < 1.5 g/dL decrease in hemoglobin, ambulation, proper voiding of urine, surgeon/anaesthesiologist	N/A	N/A	Broad selection protocol, only study to find a significant difference in admissions for patients with and without OSA, gives no further information on comorbidities between both populations,
Kleipool	Age < 65, BMI < 50, treated adhered sleep apnea, optimized comorbidities, anaesthesiologist; surgeon/anaesthesiologist, stable vital signs after 6h (<100 bpm, < 38 C, < 10 mmHg drop in diastolic blood pressure, oxygen saturation >95%) no surgical or anesthetic complications or abnormalities, < 1.0 mmol/L decrease in hemoglobin	75% Abnormal vital signs, 25% Delayed start of surgery	6.1% cardiorespiratory complications	Well defined protocol for selection and discharge protocol. Bias introduced due to assuming that unstable vital signs are due to cardiorespiratory complications, small sample size, respiratory complication defined as oxygen saturation <90% on nasal cannula, apnea lasting more than 10s, or <8 breaths per minute
Masaracchia	surgeon/anaesthesiologist; surgeon/anaesthesiologist	N/A	N/A	Used a national database to identify ambulatory surgeries, used ICD codes to identify complications, large population size, found a statistically significant difference with a multivariate analysis in unplanned admissions and complications
Melton	No severe OSA, no severe comorbidities, no BMI > 50; surgeon/anaesthesiologist	N/A	N/A	Only large population ASC study that uses multiple ASCs, strict selection criteria, no statsistically significant difference in unplanned admissions between patients with/without OSA in univariate analysis
Sabers	surgeon/anaesthesiologist; surgeon/anaesthesiologist	48.2% Surgical indication, 41.1% Pain 12.5% Nausea/Vomiting, 5.4% Episodic desaturation, 1.8% Bronchospasm 26.8% Other indication; 36.4% Surgical indications, 20.5% Pain 20.5% Nausea/Vomiting, 2.3% Ventilatory support 34.1% Other indications	1.3% Upper airway obstruction 0.4% Bronchospasm 0.4% Difficult mask ventilation; 0.4% Atrial Fibrillation 0.4% Anaphylaxis 0.4% Laryngospasm	Only study to attempt to control for patient factors, higher rate for both populations than for the general population, no statistically significant difference for unplanned admissions or complications between patients with/without OSA, low rate of respiratory complications likely due to only reporting complications that led to admissions. Only study to report specifically complications that occurred 24 hours within discharge
Scotcher	surgeon/anaesthesiologist; three consecutive stable oxygen saturation readings and surgeon/anaesthesiologist	None	None	Case-series study that found no unplanned admissions in a small population
Stierer	surgeon/anaesthesiologist, no pregnancy, no supplemental oxygen home use, no tracheostomy; Aldrete Scoring system	N/A	N/A	Used a propensity analysis, only statistically significant association for OSA propensity and supplemental oxygen to maintain above 95%, none for unplanned admissions, or arrythmias

*It was assumed to be surgeon anaesthesiologist judgement if there was not a defined selection/discharge criteria*.

### Study characteristics

The literature consisted of 30,640 patients with OSA [19–30]. Ten studies used a comparative group of 317,085 patients without OSA [19–24,26–29]. There are nine hospital studies with 9088 patients with OSA and 122,936 patients without OSA [20,22–26,28,30]. There are six retrospective cohort hospital studies [20,22,24,26,28] and one prospective cohort study [23]. There are two prospective case-series studies [25,30]. Three studies were performed in ASCs with 21,455 patients with OSA and 193,545 patients without OSA [19,27,29]. All of these studies are cohort studies. One study was prospective [29]. There is one retrospective cohort study that used data from hospitals and ASCs with 97 patients with OSA and 604 patients without OSA [21]. Identification of OSA varied across studies: 4 used polysomnography data (PSG) [20,23,25,28], 10 relied on the preoperative record or self-report [19,21–24,26,27,29,30]. Notably, one study employed multiple methods of OSA identifications [23].

### Unplanned hospital admissions

Unplanned hospital admissions for patients with OSA had a weighted average rate of 1.1%. (0.0–47.3%) [19–30]. Patients without OSA had a weighted average rate of 0.2% with less variation (<0.1–29.8%) [19–24,26–29]. ASC studies reported a 0.2% hospital admission rate [19,27,29] for patients with OSA and 0.1% unplanned admission rate for patients without OSA [19,27,29]. Three of these studies performed a statistical analysis and found no significant difference between OSA and non-OSA population [19,27,29]. Only one ASC study [19] used a selection protocol based on uncontrolled comorbidities and/or untreated sleep apnea. The other studies were assumed to rely on the surgeon/anaesthesiologist’s judgment [27,29]. Compared to ASC studies, outpatient hospital studies had higher rates of unplanned admissions for patients with OSA with a weighted average of 2.4% [20–26,28,30]. Patients without OSA had a unplanned admission rate of 0.1% in hospital studies [20,22–24,26,28]. Of these hospital studies, only three [20,23,25] used a selection protocol that took well controlled comorbidities and/or sleep apnea into consideration while the rest were up to anaesthesiologist/surgeon.

Six hospital studies statistically compared admission rates between the two groups and three found a statistically significant difference in admission rates [21,24,26]. Among these studies, Jackson et al. employed a broad selection protocol but did not use multivariate analysis to account for comorbidities as a potential cause of increased unplanned admissions [24]. Conversely, Masaracchia et al. is one of two large population studies within this review. It was the only study of its scale to incorporate a multivariate analysis considering age, sex, comorbidities, among other factors, and identified a statistically significant increase in unplanned admissions [26]. The only other study that had a population of that size was Melton et al. which incorporated a univariate analysis and found no significant difference in unplanned admissions. However, Melton et al was performed in the ASC setting.

### 24 hour postoperative complications

Five studies reported varied 24-hour postoperative complications rates [20,23,25,28,30]. All of these studies were performed in the hospital environment. One study estimated an all-complication rate of 16.0% for patients with OSA which was not statistically significant when compared to 13.1% in the non-OSA population [20]. Remaining studies reported cardiorespiratory complications for patients with OSA with a weighted average of 8.3% [23,25,28,30] with non-OSA controls having a complication rate of 12.2% [23,28].

The majority of 24 hour complications reported occurred during the PACU (within 4 hours of surgery) but 24 hour post-discharge reporting is rare among the studies. When severe complications within 24 hours were reported, patients without OSA had similar numbers of severe complications [20,28]. Moreover, reporting of cardiorespiratory complications varied across studies. Kleipool et al. only reported unstable vital signs that led to admissions, assumed as cardiorespiratory complications [25]. Hudson et al. reported all postoperative cardiorespiratory 24 hour complications [23].

### 30 day postoperative complications

Thirty day complications primarily consisted of post-discharge readmissions and revisits. Only three studies reported an overall 30-day complication rate [25,26,30]. Kleipool reports a rate of 6.1% complication rate for its population [25]. Masaracchia et al. report a significantly higher complication rate of 4.10% in the OSA population compared to 1.22% in the non-OSA population [26]. Masaracchia et al. also reported a higher rate of pulmonary compromise, myocardial infarction, pneumonia, and acute renal failure in the OSA population. Readmission rate was reported in 5 studies and had a weighted average rate of 0.3% (0.0–8.2%) [20,23,25,26]. Only one study reported ER revisits which gave a rate of 14.3% [25]. Among the studies that reported on mortality rates, only Masaracchia et al., a large population study, documented a single death in the OSA population and found no statistically significant difference in mortality rates between the OSA and non-OSA populations [20,22,23,25,29,30].

### Risk of bias

Tables 9 and 10 present the risk of bias for included studies. (S3 Data) 9 studies were analyzed using the NOS for risk of bias analysis [19–24,26–28]. Nine studies reported a 6 or 7 on the NOS with an average of 6.6, a moderate quality score. Almost all studies reported a 0/2 on comparability [19,20,22–24,27]. While many of these studies reported age, BMI, and/or AHI, only three studies considered confounding factors in its design/analysis [21,26,28]. Sabers et al. used a case-control design that considers gender, age, and BMI but not comorbidities and severity of OSA. Masaracchia et al. used a multivariate analysis that considers gender, age, BMI and comorbidities but not severity of OSA. 3 studies were analyzed using Murad’s tools [25,29,30]. Two studies were strong in all areas while Stierer et al. had weak ascertainment and selection domains.

**Table 10:** Risk of bias tool using Newcastle-Ottawa scale.

Studies	Selection	Comparability	Outcome	Total
Berend, 2018 [19]	3/4	0/2	3/3	6/9
Bryson 2012 [20]	3/4	0/2	3/3	6/9
Christian, 2019 [21]	2/4	2/2	3/3	7/9
Dupanovic, 2017 [22]	3/4	0/2	3/3	6/9
Hudson, 2018 [23]	3/4	0/2	3/3	6/9
Jackson, 2023 [24]	4/4	0/2	3/3	7/9
Masaracchia, 2018 [26]	4/4	2/2	3/3	9/9
Melton, 2020 [27]	4/4	0/2	3/3	7/9
Sabers, 2003 [28]	2/4	2/2	3/3	7/9

**Table 9:** Risk of bias tool for case series using murad tool.

Studies	Selection	Ascertainment		Causality	Reporting
Kleipool, 2023 [25]	1	1	1	1	1
Scotcher, 2023 [30]	1	1	1	1	1
Stierer, 2010 [29]	0	1	0	0.5	1

## Discussion

Concerns regarding ASCs for patients with OSA revolve around two issues: unplanned admissions and severe complications. As mentioned before, unplanned admissions present a safety risk for patients. Furthermore, the financial cost of transferring patients to hospital would negates the cost-saving purpose of ASCs. Lastly, severe complications should be preferably handled in hospital outpatient departments due to their greater resources and proximity to intensive care units.

### Unplanned admissions

Similar to Ceban et al., this review found that patients with OSA undergoing ambulatory surgery are at a higher risk of unplanned admission. While smaller population studies do not find this trend, large scale studies within this review have found significant increases of unplanned admissions [21,24,26]. Jackson et al., which employs a broad selection protocol, has shown that patients with OSA are more likely to have unplanned admissions. However, this is likely secondary to the high prevalence of comorbidities (diabetes, hypertension, obesity) in patients with OSA [31–33]. More narrow selection protocols within large population hospital studies continue to report significant increases in unplanned admissions even when accounting comorbidities with a multivariate analysis [21,26]. There are multiple physiological factors for this. The low pharyngeal tone of OSA makes these patients vulnerable to airway collapse after the use of anesthetics, opioids, and sedation [34]. Furthermore, OSA has been associated with hemodynamic instability post-operatively [7].

However, ASC studies included within our review did not find similar results. Melton et al. includes over 21 thousand patients with OSA and did not report a statistically significant higher risk of unplanned admission [27]. Furthermore, Bryson et al, a hospital study, had a similar number of patients with OSA in comparison to Christian et al. and still found no significant difference in unplanned admission [20,21]. Several factors may explain this discrepancy within our data. First, Christian et al. and Masaracchia et al. both were orthopedic surgery studies. Ceban et al found that orthopedic surgeries are associated with a higher rate of unplanned admission in comparison to other ambulatory surgeries. Melton et al and Bryson et al. included a broader range of surgeries. So, patients with OSA were less likely to undergo invasive and painful surgeries within their studies. Second, ASC studies like Melton et al. will likely have healthier OSA patients in comparison to hospital settings.. Silber et al. found similar results within their study comparing elderly patient undergoing surgery at ASCs and hospital settings [35].

### Postoperative complications

All studies that reported 24 hours complications were hospital studies. However, for every study that reported severe complications in patients, a similar rate of severe complications in 24 hours. was found in the patients without OSA. Masaracchia et al reported multiple severe complications are more likely to occur in the ambulatory surgery patient population 30 days after surgery. However, it should be noted that Masaracchia et al. get its data from hospitals and assume that all shoulder arthroplasty will be performed ambulatory. This will mean this data includes patients that were not intended to undergo outpatient surgery and more likely to have severe complications.

With appropriate patient selection and similar rates of severe complications within 24 hours, patients with OSA can safely undergo surgery in ASC environments. Patient selection is crucial to ensuring the safety of patients with OSA in ASCs. As demonstrated by Masaracchia et al., OSA remains an independent risk factor for admissions; therefore, surgeons and anesthesiologists should maintain more selective criteria when assessing comorbidities and patient characteristics. Patients with OSA selected for outpatient surgery at an ASC should have well-managed comorbidities,treated sleep apnea, and with no severe cardiovascular, respiratory, or metabolic diseases. Furthermore, it is advisable to implement perioperative interventions, including the use of CPAP machines post-operative, opioid-sparing techniques, and thorough preoperative screening for undiagnosed sleep apnea [36,37]. Additionally, it is crucial to consider specific patient characteristics associated with OSA. The literature indicates that morbid obesity and age over 65 are significant risk factors for unplanned admissions following ambulatory surgery [38,39].

Concerns of undiagnosed sleep apnea exist within this data. Literature has estimated that about 90% of sleep apnea are undiagnosed and undiagnosed sleep apnea could be a cause for why large sample sizes are needed to show differences in unplanned admissions. Bryson et al. and Hudson et al. control for this factor by using prior PSGs and using preoperative respiratory polygraphy to control for undiagnosed sleep apnea, respectively [20,23]. However, both studies were small sample size studies and found OSA did not increase unplanned admissions.

Our review has several limitations. Firstly, the one most common complication of OSA patients undergoing surgery was oxygen desaturation in PACU. However, these patients have been desaturating nightly for years pre-operatively, using this as a complication measure is likely inflating the complication rate. Secondly, there was no limitations within our inclusion criteria on selection/discharge protocol. As a result of this, we included many studies such as Jackson et al. that were not relevant to the ASC environment. Furthermore, our review lacked a meta-analysis for a more quantitative assessment of the evidence. Without data aggregation across studies, our data is less robust and should be interpreted with due caution. Further large population research is required within ASCs so that robust meta-analysis can be performed on this topic. Another significant limitation of our review was the predominance of retrospective studies which are prone to biases and errors such as selection bias. Moreover, complication reporting in many studies had unclear or incomplete data, leading to the use of surrogate measures like indications for unplanned admissions, introducing uncertainty. Additionally, it is unclear whether 30-day readmissions were a direct consequence of the surgery itself or unrelated causes and underreporting may exist as most studies only retrieve data from one hospital, overlooking patients who may have been readmitted elsewhere. The absence of detailed, and consistent complications reporting limits the reliability of our findings. The heterogeneity across study methodologies, patient populations, and surgical procedures complicates the task of drawing firm conclusions and highlights the need for standardized approach in future research. Lastly, our review is limited by the studies that are currently available; unpublished studies or unindexed studies could alter our conclusions.

## Conclusion

The review suggests a new perspective on suitability of patients with OSA in an ASC setting, emphasizing selective patient recruitment. While OSA may be an independent risk factor for unplanned admissions in ambulatory surgery, Patients with OSA with well-managed conditions can safely undergo non-upper airway surgery at an ASC.

## Supporting information

S1 Table Ovid-MEDLINE search strategy and results. (DOCX)

S2 Table Ovid-Embase search strategy and results. (DOCX)

S3 Table Scopus search strategy and results. (DOCX)

S4 Table Cochrane search strategy and results. (DOCX)

S1 File Standardized Checklist used to screen full-text articles. (DOCX)

S2 File Manual for Newcastle-Ottawa scale. (DOC)

S3 File Newcastle-Ottawa scale. (DOC)

S4 File Murad et al. tool. (DOCX)

S1 Data Excluded and included studies. (XLSX)

S2 Data Data extracted from included studies. (XLSX)

S3 Data Risk of bias from included studies. (XLSX)
