# Supplementary material for: Safety of outpatient non-upper airway surgery for patients with obstructive sleep apnea in ambulatory surgical centers: A systematic review
Source: PLoS One. 2025 Jul 7;20(7):e0326704. doi: 10.1371/journal.pone.0326704 (PMC12233240; doi:10.1371/journal.pone.0326704)
Supplement: S1 Table — (DOCX) [file pone.0326704.s001.docx]

**S1 Table :** Ovid-MEDLINE Search Results

| Query | Search Terms | Results |
| --- | --- | --- |
| #1 | Sleep Apnea Syndromes/ OR Sleep Apnea, Obstructive/ OR sleep apnea.mp. OR osa.mp. OR sleep apnoea.mp. OR osahs.mp. OR osas.mp. | 59,748 |
| #2 | Ambulatory Surgical Procedures/ OR Outpatients/ OR Surgicenters/ OR Ambulatory Care/ OR Ambulatory Care Facilities/ OR Ambulatory Care Sensitive Conditions/ OR ambulatory.mp. OR outpatient.mp. OR same day surgery.mp. OR same day discharge.mp. OR ambulatory surgical center.mp. OR ambulatory surgery center.mp. OR hopd.mp. OR day case surgery | 337,671 |
| #3 | #1 AND #2 | 2495 |
