# Supplementary material for: Safety of outpatient non-upper airway surgery for patients with obstructive sleep apnea in ambulatory surgical centers: A systematic review
Source: PLoS One. 2025 Jul 7;20(7):e0326704. doi: 10.1371/journal.pone.0326704 (PMC12233240; doi:10.1371/journal.pone.0326704)
Supplement: S2 Table — (DOCX) [file pone.0326704.s002.docx]

**S2 Table:** Ovid-Embase Search Results

| Query | Search Terms | Results |
| --- | --- | --- |
| #1 | sleep disordered breathing/ OR upper airway resistance syndrome/ OR sleep apne*.mp.OR sleep apnoe*.mp OR (obstruct* adj2 hypopnea*).mp. OR (obstruct* adj2 hypopnoea*).mp. OR (obstruct* adj2 hypoapnea*).mp OR (obstruct* adj2 hypoapnoea*).mp OR (sleep disorder* adj1 breathing).mp OR osa.mp OR osas.mp. OR osahs.mp. | 115,385 |
| #2 | ambulatory surgery/ OR outpatient care/ OR outpatient/ OR outpatient department/ OR outpatient.mp. OR (same day adj2 surg*).mp. OR ambulatory.mp. OR day surgery.mp. OR (day-case adj2 surg*).mp. OR (same-day adj2 discharg*).mp. | 595,339 |
| #3 | #1 AND #2 | 5435 |
| #4 | limit #3 to "remove medline records" | 2924 |
