# Supplementary material for: Safety of outpatient non-upper airway surgery for patients with obstructive sleep apnea in ambulatory surgical centers: A systematic review
Source: PLoS One. 2025 Jul 7;20(7):e0326704. doi: 10.1371/journal.pone.0326704 (PMC12233240; doi:10.1371/journal.pone.0326704)
Supplement: S3 Table — (DOCX) [file pone.0326704.s003.docx]

**S3 Table:** Scopus Search Results

| Query | Search Terms | Results |
| --- | --- | --- |
| #1 | ( TITLE-ABS-KEY ( "sleep apne*" ) OR TITLE-ABS-KEY ( "osa" ) OR TITLE-ABS-KEY ( "osas" ) OR TITLE-ABS-KEY ( "osahs" ) OR TITLE-ABS-KEY ( "obstruct*" W/2 "hypopnea*" ) OR TITLE-ABS-KEY ( "obstruct*" W/2 "hypopnoea*" ) OR TITLE-ABS-KEY ( "obstruct*" W/2 "hypoapnea*" ) OR TITLE-ABS-KEY ( "obstruct*" W/2 "hypoapnoea*" ) OR TITLE-ABS-KEY ( "obstruct*" W/2 "hypo-apnea*" ) OR TITLE-ABS-KEY ( "obstruct*" W/2 "hypo-apnoea*" ) OR TITLE-ABS-KEY ( "sleep disorder*" W/1 "breathing" ) OR TITLE-ABS-KEY ( "sleep apno*" ) ) AND (( TITLE-ABS-KEY ( "ambulatory" ) OR TITLE-ABS-KEY ( "outpatient" ) OR TITLE-ABS-KEY ( "day surgery" ) OR TITLE-ABS-KEY ( "day-case" ) OR TITLE-ABS-KEY ( "same day" W/2 "surg*" ) OR TITLE-ABS-KEY ( "same-day" W/2 "discharg*" ) OR TITLE-ABS-KEY ( "day case" ) OR TITLE-ABS-KEY ( "daycase" ) OR TITLE-ABS-KEY ( "day patient" ) ) .  ( ( TITLE-ABS-KEY ( "sleep apne*" ) OR TITLE-ABS-KEY ( "osa" ) OR TITLE-ABS-KEY ( "osas" ) OR TITLE-ABS-KEY ( "osahs" ) OR TITLE-ABS-KEY ( "obstruct*" W/2 "hypopnea*" ) OR TITLE-ABS-KEY ( "obstruct*" W/2 "hypopnoea*" ) OR TITLE-ABS-KEY ( "obstruct*" W/2 "hypoapnea*" ) OR TITLE-ABS-KEY ( "obstruct*" W/2 "hypoapnoea*" ) OR TITLE-ABS-KEY ( "obstruct*" W/2 "hypo-apnea*" ) OR TITLE-ABS-KEY ( "obstruct*" W/2 "hypo-apnoea*" ) OR TITLE-ABS-KEY ( "sleep disorder*" W/1 "breathing" ) OR TITLE-ABS-KEY ( "sleep apno*" ) ) AND ( TITLE-ABS-KEY ( "ambulatory" ) OR TITLE-ABS-KEY ( "outpatient" ) OR TITLE-ABS-KEY ( "day surgery" ) OR TITLE-ABS-KEY ( "day-case" ) OR TITLE-ABS-KEY ( "same day" W/2 "surg*" ) OR TITLE-ABS-KEY ( "same-day" W/2 "discharg*" ) OR TITLE-ABS-KEY ( "day case" ) OR TITLE-ABS-KEY ( "daycase" ) OR TITLE-ABS-KEY ( "day patient" ) ) ) | 3721 |
