# Supplementary material for: Safety of outpatient non-upper airway surgery for patients with obstructive sleep apnea in ambulatory surgical centers: A systematic review
Source: PLoS One. 2025 Jul 7;20(7):e0326704. doi: 10.1371/journal.pone.0326704 (PMC12233240; doi:10.1371/journal.pone.0326704)
Supplement: S4 Table — (DOCX) [file pone.0326704.s004.docx]

**S4 Table**: Cochrane Search Results

| Query | Search Terms | Results |
| --- | --- | --- |
| #1 | MeSH descriptor: [Sleep Apnea Syndromes] explode all trees | 3793 |
| #2 | "sleep apnea" OR "sleep apnea hypopnea syndrome" OR "sleep apnea-hypopnea syndrome" or "sleep apnea syndrome" OR "sleep-disordered breathing" OR "sleep-disordered breathing" OR "sleep apnoea*" OR "sleep apnoea hypopnoea syndrome" OR "sleep apnoea syndrome" OR "sleep apnoea-hypopnoea syndrome" OR "OSA" OR "OSAS" OR "OSAHS" | 9433 |
| #3 | obstruct* NEAR/2 "hypopnea" | 95 |
| #4 | obstruct* NEAR/2 hypopnoea | 92 |
| #5 | MeSH descriptor: [Ambulatory Surgical Procedures] explode all trees | 1717 |
| #6 | MeSH descriptor: [Outpatients] this term only | 1877 |
| #7 | MeSH descriptor: [Ambulatory Care Facilities] this term only | 764 |
| #8 | MeSH descriptor: [Ambulatory Care] this term only | 3562 |
| #9 | MeSH descriptor: [Surgicenters] this term only | 8 |
| #10 | "ambulatory" OR "outpatient" OR "same day surgery" OR "same-day surgery" OR "daycase" OR "day-case" OR "day-case" | 64,831 |
| #11 | "same day" NEAR/2 discharg* OR "day-case" NEAR/2 surg* OR "same day" NEAR/2 surg* | 908 |
| #12 | #1 OR #2 OR #3 OR #4 | 9478 |
| #13 | #5 OR #6 OR #7 OR #8 OR #9 OR #10 OR #11 | 65,636 |
| #14 | #12 AND #13 | 667 |
| #15 | Selected only CENTRAL trials from #14 | 591 |
