# Supplementary material for: Safety of outpatient non-upper airway surgery for patients with obstructive sleep apnea in ambulatory surgical centers: A systematic review
Source: PLoS One. 2025 Jul 7;20(7):e0326704. doi: 10.1371/journal.pone.0326704 (PMC12233240; doi:10.1371/journal.pone.0326704)
Supplement: S1 File — (DOCX) [file pone.0326704.s005.docx]

**Standardized Checklist to Determine Inclusion of Study**

Inclusion

1. >10 patients with OSA determined by polysomnography, patient self-report, and preoperative records
2. Adult patients (>18 years old)
3. Undergoing on-upper airway same-day discharge ambulatory surgery with >50% using general anesthesia
4. Must include unplanned admission

Exclusion

1. Non-original research
2. Pediatric studies
3. Questionnaires
4. Cardiovascular surgery
5. Animal subjects
6. Non-english
