# Supplementary material for: Safety of outpatient non-upper airway surgery for patients with obstructive sleep apnea in ambulatory surgical centers: A systematic review
Source: PLoS One. 2025 Jul 7;20(7):e0326704. doi: 10.1371/journal.pone.0326704 (PMC12233240; doi:10.1371/journal.pone.0326704)
Supplement: S4 File — (DOCX) [file pone.0326704.s008.docx]

**MURAD ET AL. SCALE FOR CASE-SERIES**

**Selection**

1. Does the patient(s) represent(s) the whole experience of the investigator (centre) or is the selection method unclear to the extent that other patients with similar presentation may not have been reported?

**Ascertainment:**

1. Was the exposure adequately ascertained?
2. Was the outcome adequately ascertained?

**Causality**

1. Was follow-up long enough for outcomes to occur?

**Reporting**

1. Is the case(s) described with sufficient details to allow other investigators to replicate the research or to allow practitioners make inferences related to their own practice?
